# Uncommon Histopathological Subtypes and Variants of Cervical Carcinoma Diagnosed at a Tertiary Care Centre: A Case Series

**DOI:** 10.7759/cureus.66783

**Published:** 2024-08-13

**Authors:** Veda Samhitha N. S., Divya Dhanabal, Sandhya Sundaram, Pavithra V., Subalakshmi Balasubramanian

**Affiliations:** 1 Pathology, Sri Ramachandra Institute of Higher Education and Research, Chennai, IND

**Keywords:** cervical biopsy, synaptophysin, serous carcinoma, basaloid variant of squamous cell carcinoma, p16 testing, post-coital bleeding, per vaginal bleeding, post-menopausal bleed, human papillomavirus (hpv), cervical carcinoma

## Abstract

Introduction

Cervical cancer ranks among the top gynaecological cancers worldwide. It is linked to lower socioeconomic status and high human papillomavirus (HPV) prevalence. This is a series of six cervical carcinoma cases analysed from 2021 to 2023 at our tertiary care centre to identify rare subtypes of cervical carcinoma. We document rare subtypes, which include glassy cell carcinoma, small cell neuroendocrine carcinoma, papillary squamous-transitional variant, basaloid squamous cell carcinoma and serous carcinoma of the uterine cervix. Immunohistochemistry (IHC) was helpful in confirmation of the subtypes and in diagnosing HPV-associated cases.

Materials and methods

This case series comprises six cases, including rare subtypes and variants of cervical carcinoma histopathologically diagnosed by the Department of Pathology, Sri Ramachandra Institute of Higher Education and Research, Chennai, India, between 2021 and 2023. The demographic profile and patient details were obtained from the hospital information system and archival case files after obtaining informed consent from the patients. The H&E and relevant IHC slides along with histopathology reports of the included cases were analysed and studied.

Results

This series includes six cases of rare subtypes of cervical carcinoma, comprising glassy cell carcinoma, small cell neuroendocrine carcinoma, papillary squamous-transitional variant, basaloid squamous cell carcinoma (SCC), and serous carcinoma. Each subtype displays distinct clinicopathological features, emphasizing the need for specific diagnostic and treatment approaches, which are crucial in improving patient survival.

Conclusion

Six rare subtypes and variants of cervical carcinoma have been discussed in this case series, after correlating with histopathology reports and clinical and radiological findings. Understanding the histopathological characteristics of these rarer subtypes is essential for accurate diagnosis and timely intervention. This series highlights the importance of comprehensive screening strategies, early diagnosis and awareness of rarer subtypes and variants of cervical carcinoma among healthcare professionals. These factors can tailor therapeutic options and improve patient outcomes.

## Introduction

Cervical carcinoma accounts for around 6,60,000 cases diagnosed worldwide, with 3,50,000 deaths, as per WHO 2022. Middle- and low-income countries contribute to most of the cases and higher mortality rates. Differences in incidence and mortality rates in each region might be due to inadequate screening programs, vaccination, treatment strategies and various risk factors among individuals. Untreated HPV-associated cervical infection may lead to cervical carcinoma in 95% of cases [1]. The most common subtype is squamous cell carcinoma (90%), followed by adenocarcinoma [2-4]. This case series comprises six cases of rare and uncommon variants and subtypes of cervical carcinoma diagnosed in the Department of Pathology at Sri Ramachandra Institute of Higher Education and Research in the period between 2021 and 2023. Early recognition of unconventional subtypes of cervical carcinoma keeps a check on the five-year survival rate and has prognostic significance.

## Materials and methods

This is a case series of six cervical carcinoma cases conducted in the Department of Pathology, Sri Ramachandra Institute of Higher Education and Research, a tertiary care hospital based in southern India during the period between January 2021 and December 2023 after obtaining informed consent from the patients.

Cervical biopsy and hysterectomy specimens of patients who presented with chief complaints including postmenopausal bleeding, post-coital bleeding and per vaginal bleeding, sent by the Department of Obstetrics and Gynaecology of Sri Ramachandra Institute of Higher Education and Research for histopathological diagnosis were considered.

Hematoxylin and eosin (H&E) staining and relevant immunohistochemistry (IHC) were performed to establish a definitive diagnosis. Non-neoplastic lesions, benign lesions, conventional and common histologic types such as squamous cell carcinoma NOS (not otherwise specified), adenocarcinoma of the cervix NOS, malignant cases post-chemotherapy, and metastatic deposits from other organs were excluded from this case series.

H&E and IHC slides for the remaining cervical carcinoma cases, including the glassy cell variant of adenosquamous carcinoma, basaloid squamous cell carcinoma (BSCC), neuroendocrine carcinoma, serous carcinoma, and two cases of papillary squamotransitional carcinoma of the cervix, were examined after obtaining informed consent from the respective patients. This study was conducted prospectively in the Department of Pathology. Medical record details and radiological findings for these cases were collected from the hospital information system. Microscopic analysis of the histopathology slides and relevant ancillary studies were documented using a light microscope (LX500) manufactured by Labomed, Gurgaon, India.

In correlation with the histopathological diagnosis and clinical and radiological findings, these six cases were compiled for the case series.

## Results

Case 1: Glassy cell carcinoma (GCC)

**Figure 1:**
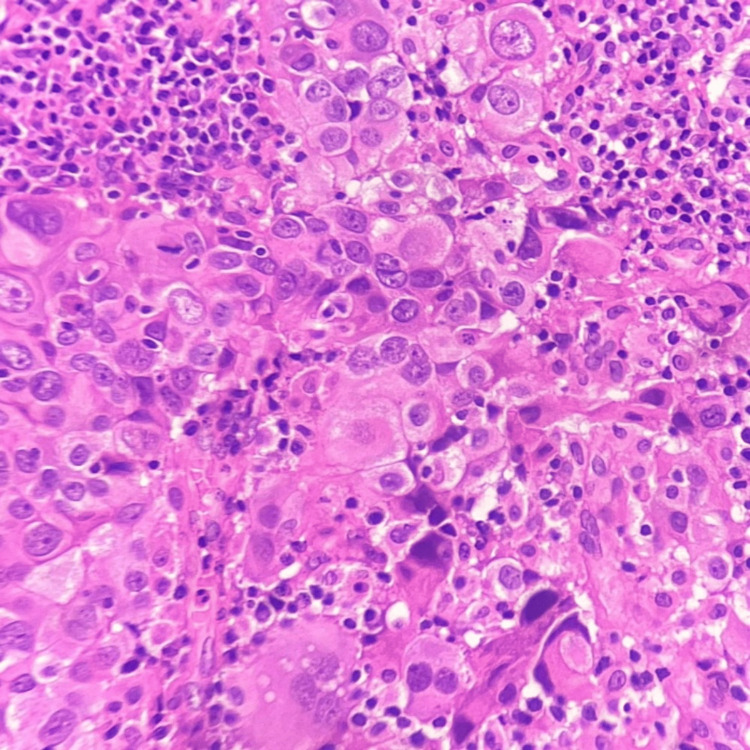
Glassy cell carcinoma showing tumour cells with ground glass cytoplasm (H&E, 400X magnification).

A 53 years old multiparous woman came with the chief complaint of bleeding per vagina. Cervical growth was noted during per speculum examination, for which a colposcopic biopsy was taken. MRI of the pelvis showed uterine myometrium and cervix infiltrated with a mass lesion measuring 5.6 x 10.5 x 4.7 cm (anteroposterior (AP) x transverse (TR) x craniocaudal (CC)). Multiple heterogeneously enhancing conglomerated metastatic deposits were noted in the pouch of Douglas, along with a few relatively well-defined T2 hyperintense lesions showing diffusion restriction noted in segments VII, I and IVA of the liver, suggestive of hepatic metastasis were also noted on plain and Contrast MRI of abdomen and pelvis. On histopathology, sections from cervical biopsy showed tumour cells arranged predominantly in sheets and focally in nests. The tumour cells are polygonal in shape with marked pleomorphism, prominent cell membranes, and moderate eosinophilic ground glass-like cytoplasm, and many cells show prominent nucleoli with surrounding inflammatory exudate (Figure 1). Many atypical mitotic figures were also noted.

**Figure 2:**
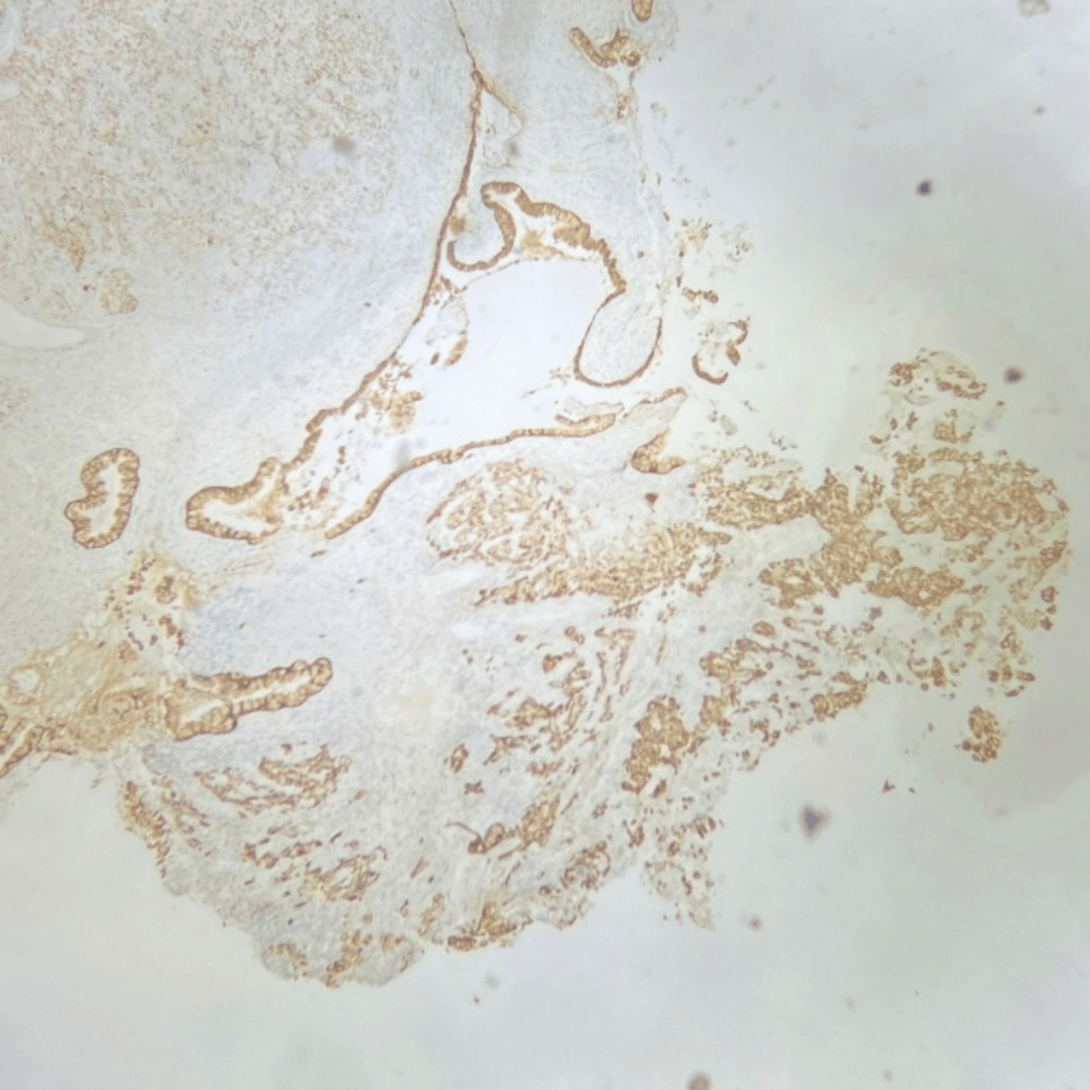
Tumour cells are positive for cytokeratin 7 (IHC, 100X magnification). IHC: immunohistochemistry

**Figure 3:**
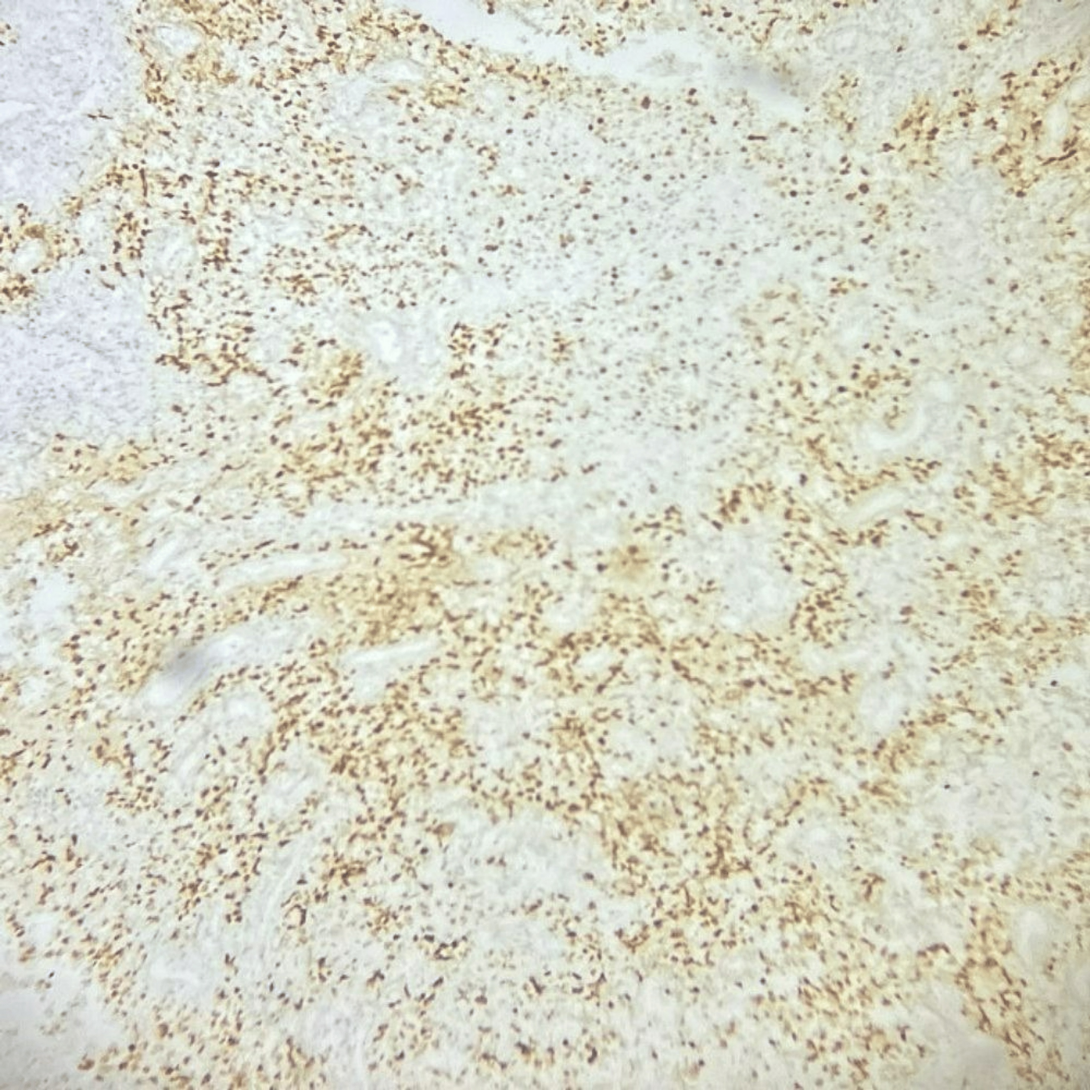
Tumour cells are positive for p63 (IHC, 100X magnification). IHC: immunohistochemistry

**Figure 4:**
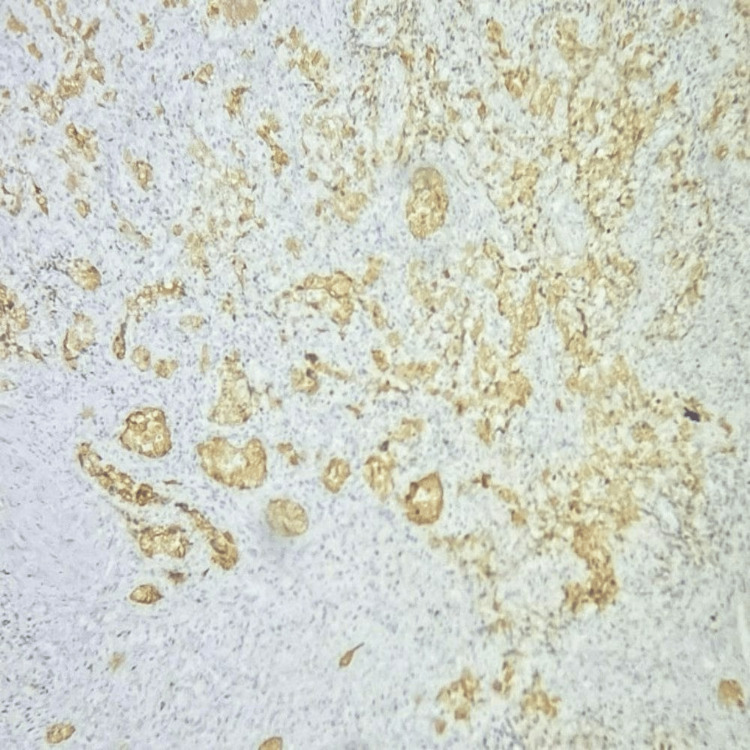
Tumour cells are positive for p16 (IHC, 100X magnification). IHC: immunohistochemistry

By IHC, the tumour cells were positive for cytokeratin and p63, and p16 showed block-like positivity (Figures 2, 3, 4). The tumour cells were focally positive for p40.

Due to distant organ spread in radiology stage IVB was assigned. The patient received five cycles of palliative chemotherapy but the MRI performed after a period of five months showed a significant increase in the size of the pelvic metastatic deposits and the cervical lesion. The patient was lost to follow up after this.

Case 2: BSCC of the cervix (BSCC)

**Figure 5:**
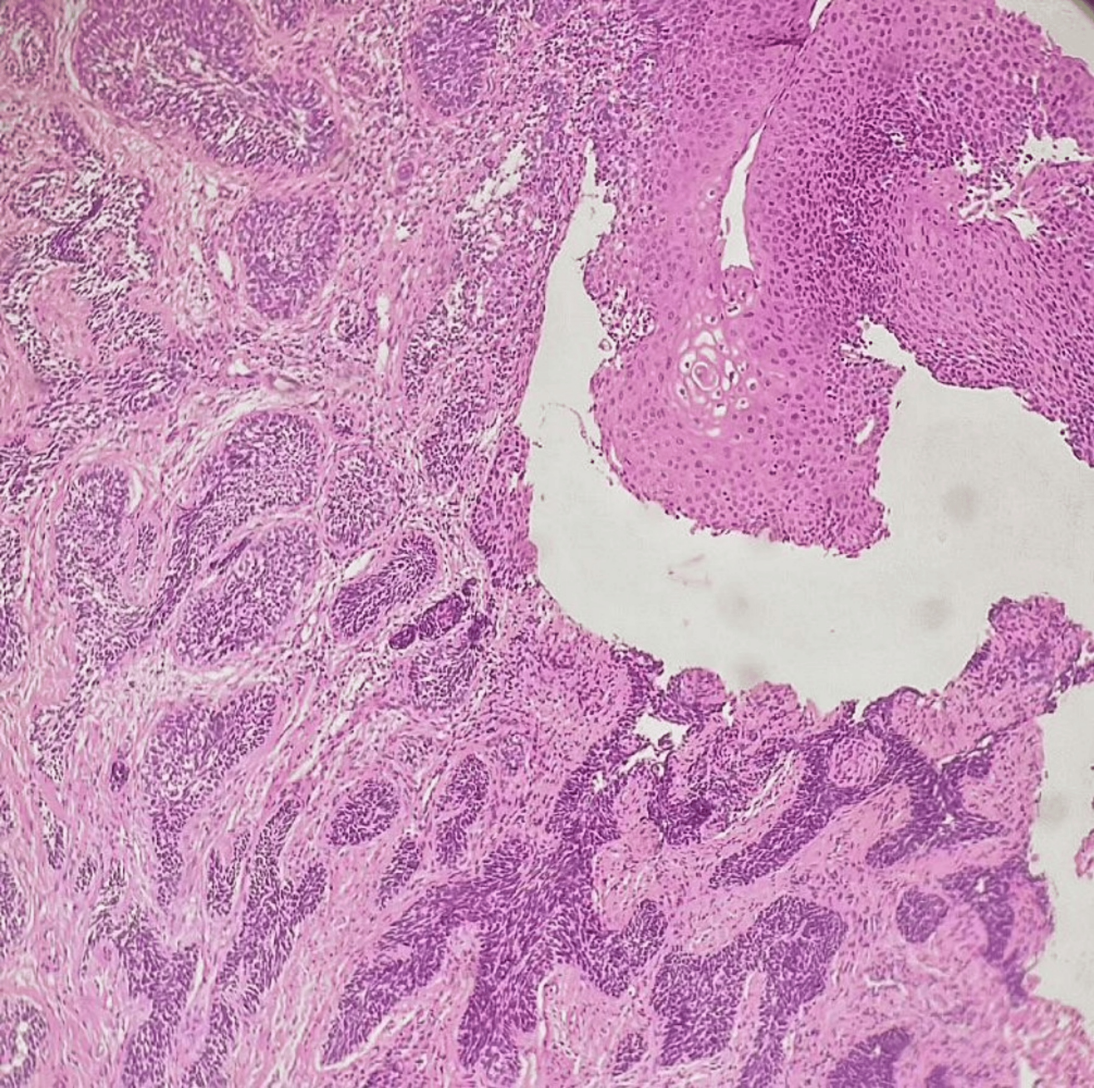
Basaloid squamous cell carcinoma of the cervix (H&E, 100X magnification).

A 42-year-old woman presented with complaints of fatigue and post-coital bleeding. MRI pelvis with abdomen screening showed an ill-defined circumferential T2 hyperintense T1 isointense lesion with restricted diffusion measuring 4.7 x 4.7 x 7 cm (AP x TR x CC) seen involving the cervix. Few hyperintense lesions were also noted in the liver and spine, likely suggestive of metastatic deposits. A punch biopsy was taken from the cervical mass. Microscopically, ectocervical and endocervical tissue were noted, with an adjacent fragment showing infiltration arranged in nested and trabecular patterns with central comedo-type necrosis and areas of keratinization. The individual tumour cells are basaloid with indistinct cell borders and vesicular nuclear chromatin, with many showing prominent nucleoli and many brisk atypical mitotic figures (Figure 5).

**Figure 6:**
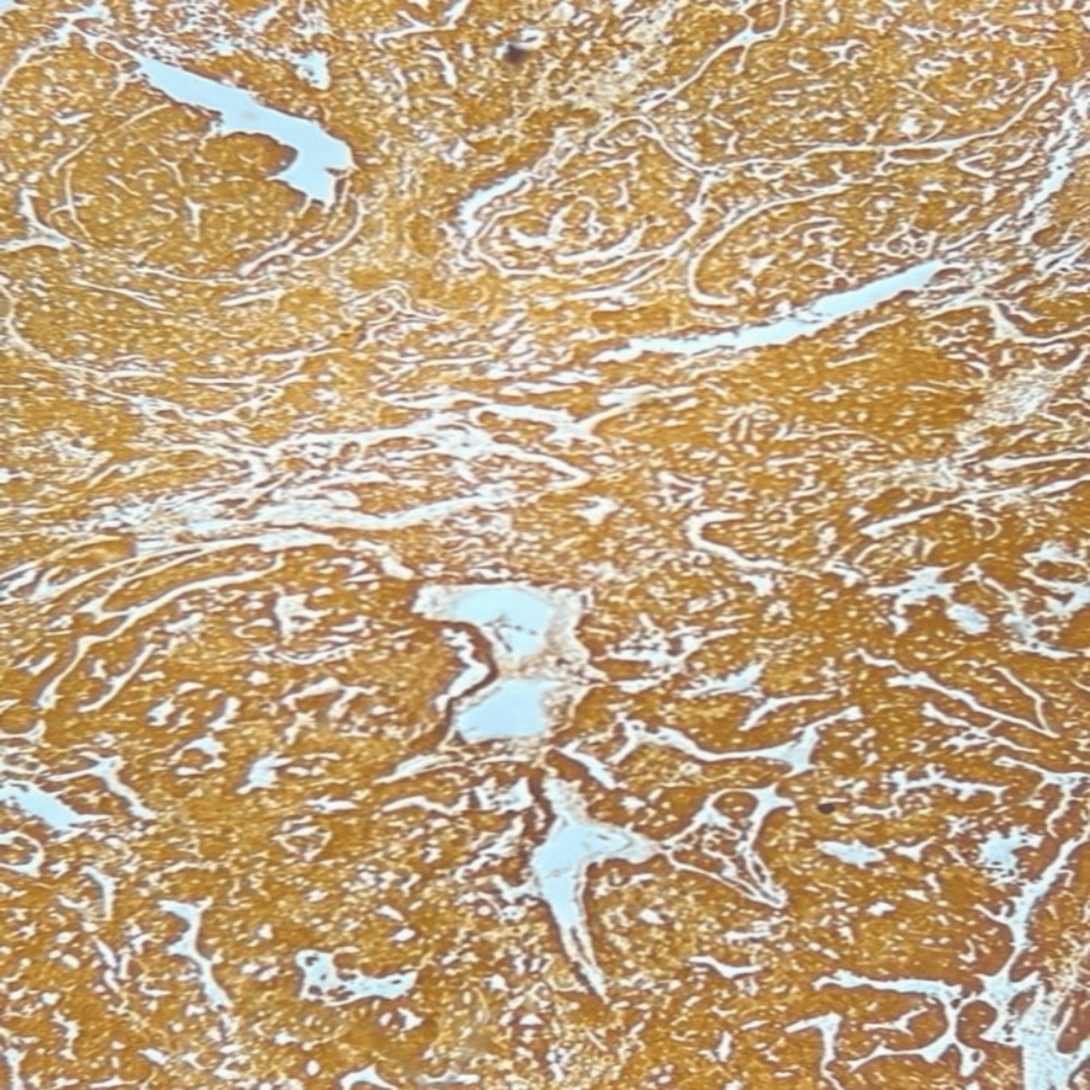
Tumour cells are positive for p16 (IHC, 100X magnification). IHC: immunohistochemistry

By IHC, tumour cells were positive for p16 (Figure 6).

Palliative chemotherapy and maintenance capecitabine were given for three months. Following this, the patient was lost to follow-up.

Cases 3 and 4: Papillary squamotransitional cell carcinoma (PSCC)

**Figure 7:**
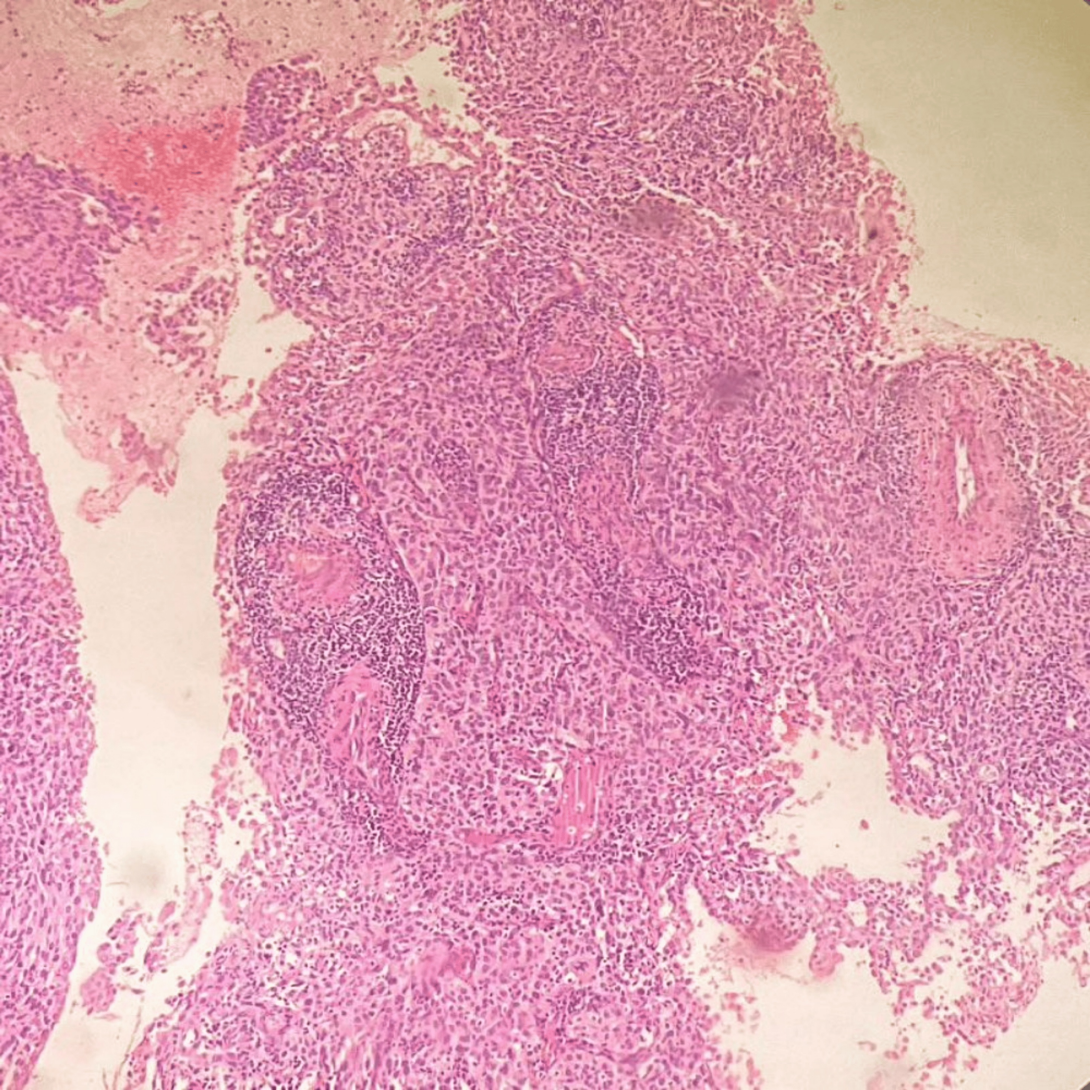
Papillary squamous-transitional cell carcinoma of the cervix (H&E, 100X magnification).

A 45-year-old woman came with the chief complaint of vaginal bleeding. MRI of the pelvis showed an ill-defined T2 hyperintense lesion showing diffusion restriction and a few areas of blooming, measuring 3.3 x 4.1 x 4.3 cm (AP x TR x CC) in the cervix, predominantly involving the anterior lip, posterior lip and left lateral wall of cervix, showing heterogeneous enhancement on contrast administration. A cervical biopsy was done, and on microscopy, tumour tissue fragments arranged in papillary architecture showed stratification, loss of polarity, pleomorphism, cytological atypia and moderate to scant cytoplasm. Brisk atypical mitosis and focal desmoplastic stromal reaction were also noted. Another case was that of a 65-year-old woman who also came with a similar chief complaint. A cervical biopsy on microscopy showed tumour tissue in a papillary pattern with central fibrovascular cores that had brisk mitoses (Figure 7).

The 45-year-old patient received adjuvant chemotherapy and radiotherapy. A radical hysterectomy specimen was received after five months. The cervix was all embedded and studied. Microscopically, no evidence of residual tumour was noted. The 65-year-old patient was lost to the follow-up post-biopsy diagnosis.

Case 5: Small cell neuroendocrine carcinoma (SCNEC) of the cervix

**Figure 8:**
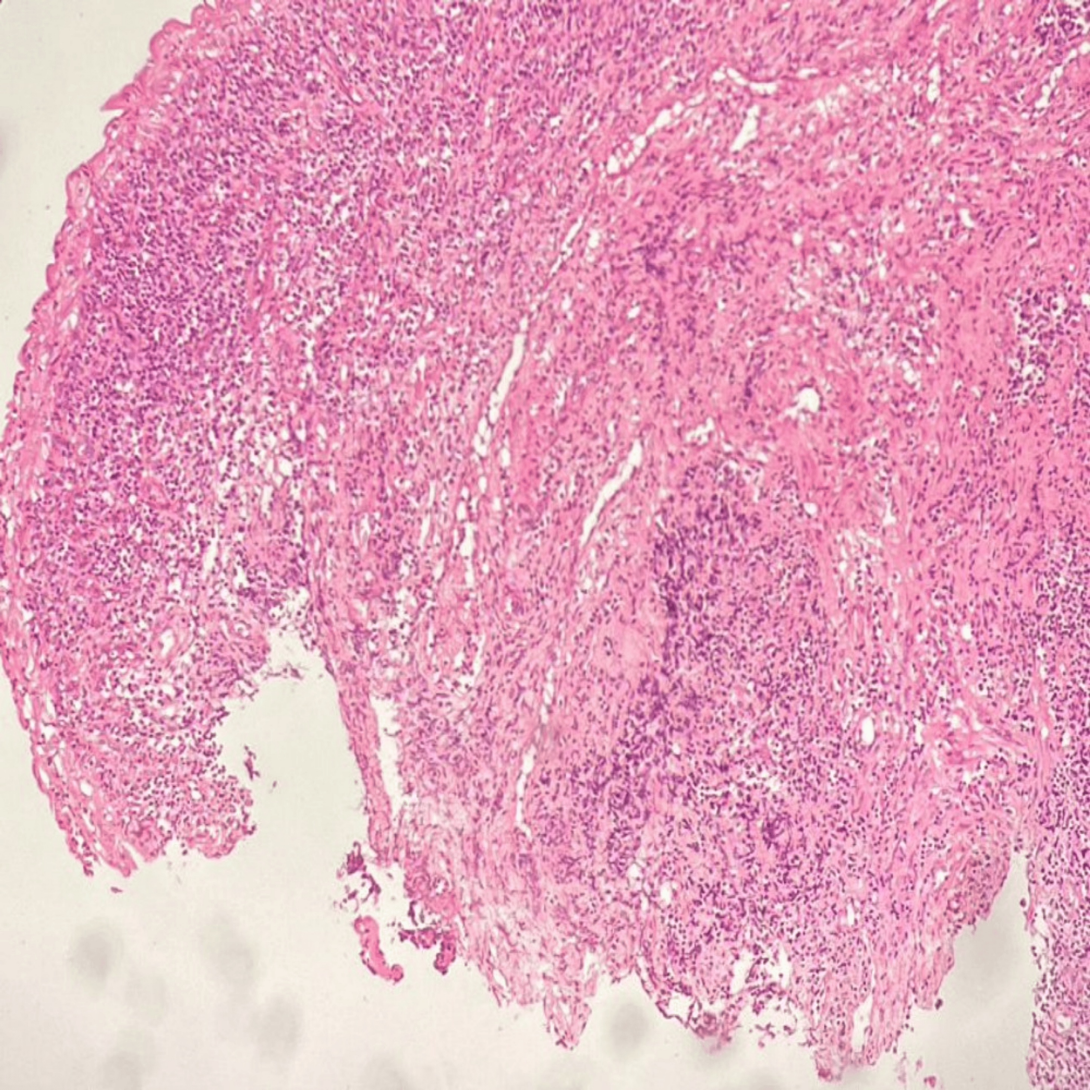
Cervical tissue infiltrated by uniform small blue tumour cells (H&E, 100X magnification).

**Figure 9:**
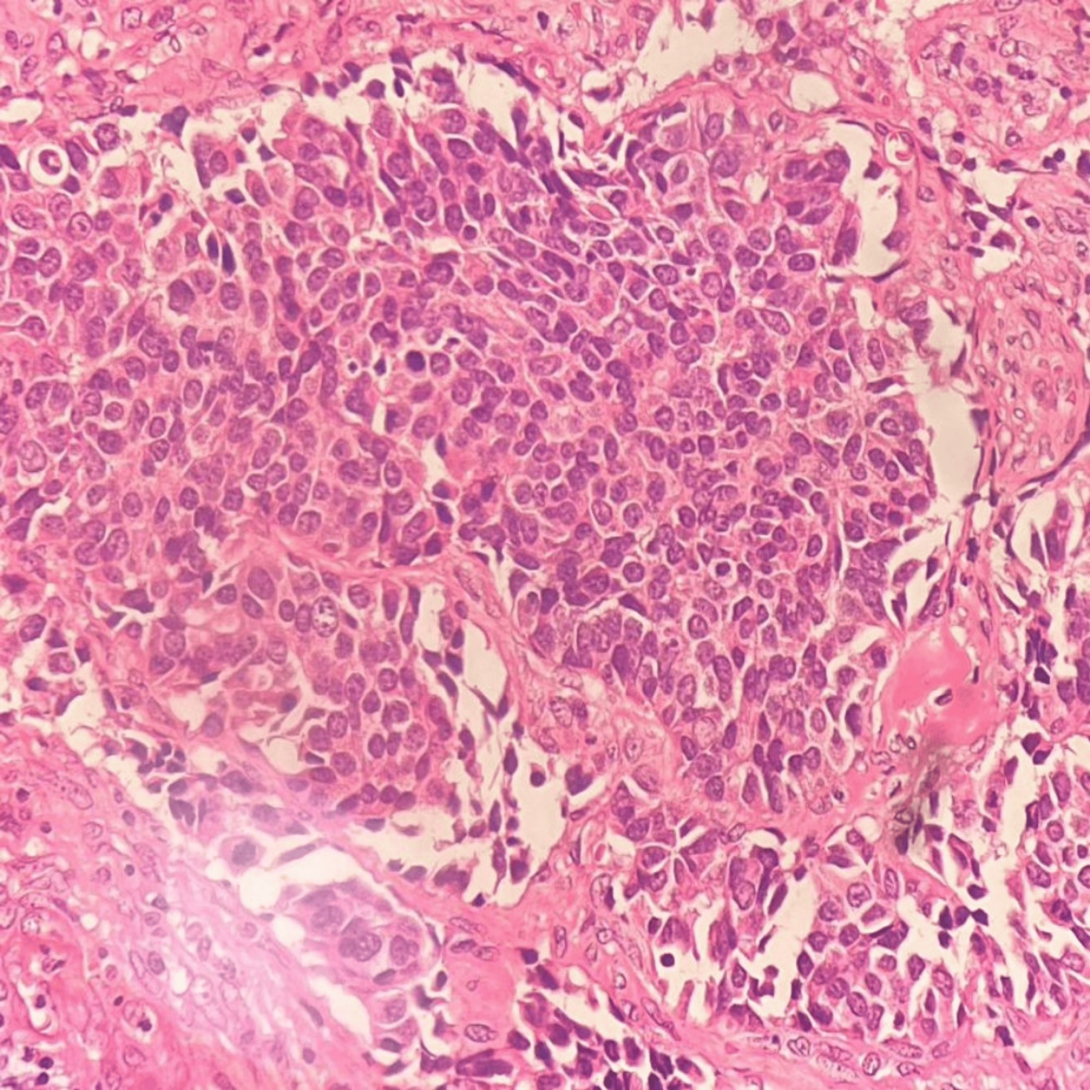
Small cell carcinoma of the cervix (H&E, 400X magnification).

A 46-year-old female presented with complaints of postcoital bleeding. On the MRI pelvis, an irregular enhancing, T2 heterogeneously hypointense, T1 isointense mass lesion measuring 3.3 x 3.5 x 3.5 cm (AP x TR x CC) was seen involving the cervix and lower uterine segment. A cervical biopsy was done. Microscopy showed tissue lined by stratified squamous epithelium with underlying stroma exhibiting loose aggregates of uniform small cells with an indistinct cell border, scant cytoplasm, hyperchromatic nuclei with granular chromatin, nuclear moulding, and indistinct nucleoli (Figures 8-9). Occasional mitotic figures were also noted.

**Figure 10:**
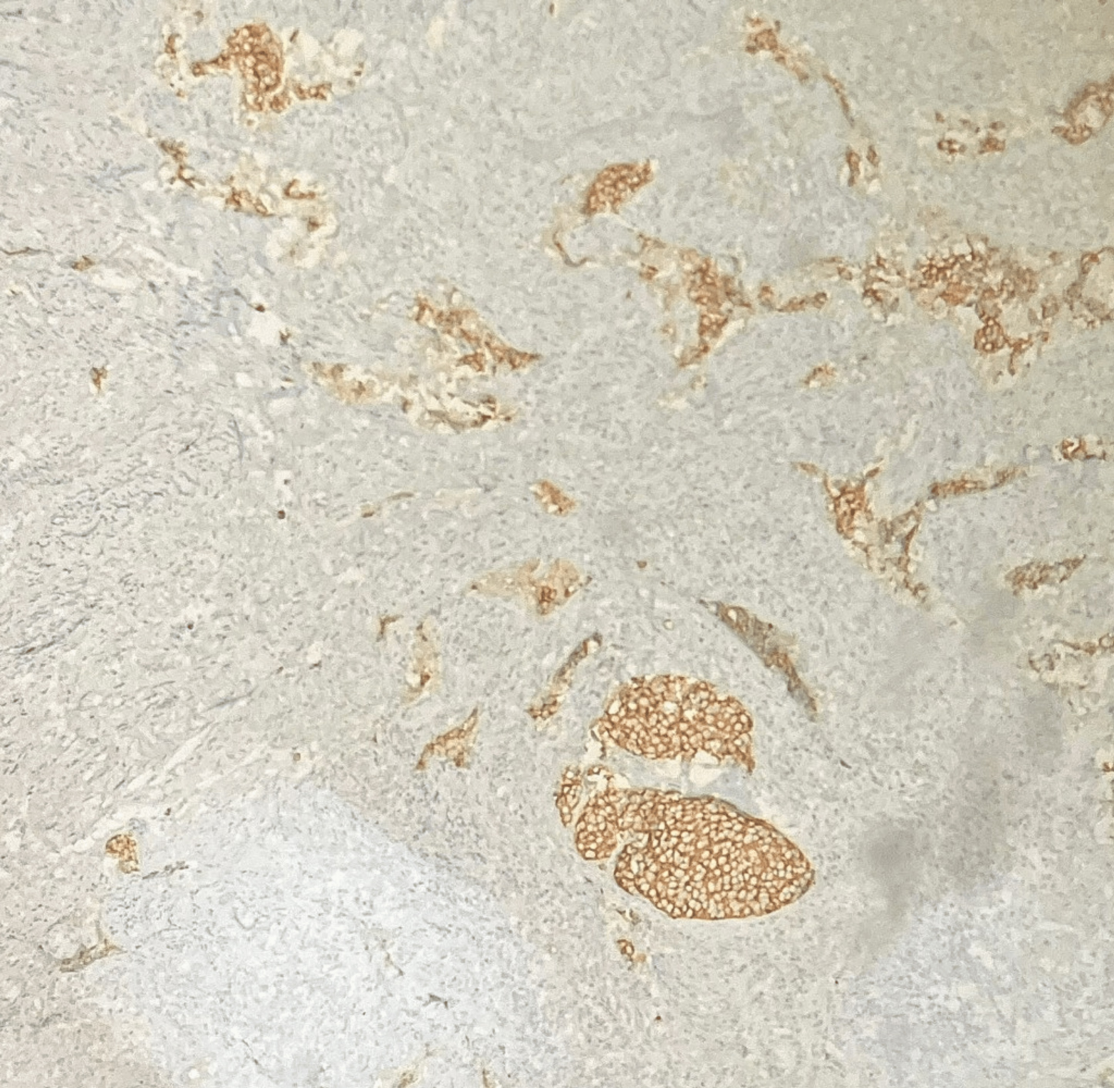
Tumour cells are positive for synaptophysin (IHC, 100X magnification). IHC: immunohistochemistry

IHC showed the tumour cells positivity for synaptophysin, CD56, PanCK and p16 (Figure 10). The tumour cells were negative for p40. ki67 labelling index was 98% in tumour cells. Hence, H&E and IHC features were that of an HPV-associated SCNEC of the cervix.

MRI pelvis done five months post-chemotherapy showed a reduction in the size of the cervical lesion to 2.3 x 2.5 x 2.5 cm (AP x TR x CC). After six months, a radical hysterectomy was done. Grossly upon embedding the cervix, no residual tumour was noted on microscopy. The postoperative period has been uneventful.

Case 6: Serous carcinoma of the uterine cervix (USCC)

A 58-year-old female presented with postmenopausal bleeding. MRI abdomen and pelvis showed an irregular mass lesion appearing hetero-intense on the T2-weighted sequence, with associated diffusion restriction seen circumferentially involving the cervix and lower uterine segment, measuring 5.2 x 4.7 x 5.9 cm extending into the upper 60% of the vagina with minimal infiltration into bilateral parametrium. A prominent lymph node was noted along the left internal iliac region measuring 7.5 mm. No other mass lesions or fluorodeoxyglucose (FDG)-avid lesions were noted.

**Figure 11:**
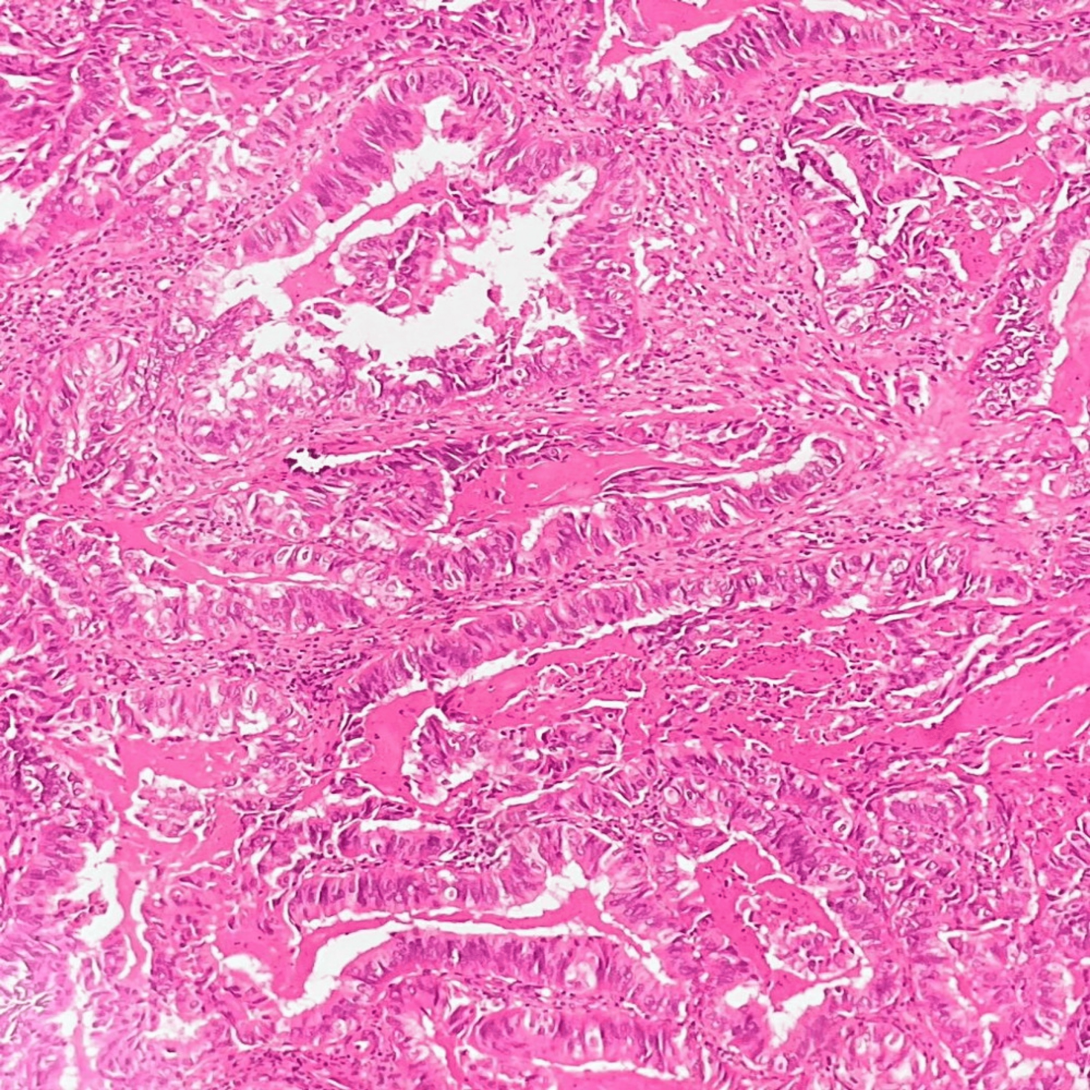
High-grade serous carcinoma of the cervix (H&E, 100X magnification).

A cervical biopsy was done, and microscopy showed tumour cells arranged in nests and papillary architecture. The individual tumour cells show nuclear pleomorphism and vesicular nuclear chromatin, and many areas show psammoma bodies (Figure 11). By IHC, the tumour cells were positive for estrogen receptor (ER), progesterone receptor (PR), p16, vimentin, and mutant p53 expression. They were negative for carcinoembryonic antigen (CEA).

Following this, the patient was lost to follow-up.

During the period between 2021-2023, six cases of rare subtypes and variants of cervical carcinoma were diagnosed (Table 1).

**Table 1:** Details of the rare cervical carcinoma cases, highlighting H&E and IHC findings. IHC: immunohistochemistry; H&E: hematoxylin and eosin; PanCK: pancytokeratin; ER: estrogen receptor; PR: progesterone receptor; CEA: carcinoembryonic antigen

S. no.	Case summary	Histopathological diagnosis	IHC
1.	53 years/multiparous female with per vaginal bleeding	Glassy cell variant of adenosquamous carcinoma	PanCK, p63: Positive; p16: Block-like positivity; p40: Focal positivity
2.	42 years/multiparous female with post-coital bleeding	Basaloid squamous cell carcinoma	p16: Positive
3.	45 years/multiparous female with per vaginal bleeding	Papillary squamotransitional cell carcinoma	-
4.	65 years/multiparous female with per vaginal bleeding	Papillary squamotransitional cell carcinoma	-
5.	46 years/multiparous female with post-coital bleeding	Small cell neuroendocrine carcinoma of the cervix	PanCK, Synaptophysin, CD56, p16: Positive
6.	58 years/multiparous female with post-menopausal bleeding	Serous carcinoma of the uterine cervix	ER, PR, p16, Vimentin: Positive; p53: Mutant expression; CEA: Negative

## Discussion

Case 1: GCC

GCC of the cervix is a distinct variant of poorly differentiated adenosquamous carcinoma. It usually affects younger patients, is often associated with pregnancy, and presents as a promptly growing mass with the shape of the cervix appearing like that of a barrel. Our patient had presented at a later age when compared to the study by Wang et al., which showed the median age of all cervical GCC patients was 46 years (range from 33 to 69 years) [5]. The patient’s demographic and clinicopathological parameters were in concordance with the study by Wang et al., which showed that most patients were multiparous women who presented with abnormal vaginal bleeding, and HPV association was seen in 44.4% of the cases. Grabowska et al. reported a case of a 26-year-old patient in her first pregnancy, at 18 weeks of gestation, admitted to the hospital due to vaginal bleeding [6]. IHC positivity for cytokeratin, p63 and p16 was noted. Unlike Grabowska et al. [6], this case showed no association between GCC of the cervix and pregnancy. Microscopically, it features large polygonal cells with ground glass-like cytoplasm, definite cell borders, vesicular chromatin, and distinct nucleoli. Mitotic figures are common, and stroma infiltration includes lymphocytes, plasma cells, and eosinophils. While lacking glycogen, it may exhibit keratinisation with squamoid or adenomatous differentiation and occasional clear cells. Ultrastructural findings suggest both squamous and glandular elements. HPV types 18 and 16 DNA have been detected. The differentials include adenocarcinoma, which usually has no mixing of tumour elements, and large cell nonkeratinizing squamous cell carcinoma, which has a less well-defined cell membrane and coarser chromatin. In a study by Zhu et al., GCC expressed markers for both squamous cell carcinoma (p63 and CK34βE12) and adenocarcinoma (CAM5.2, MUC1, MUC2 and CEA) while the Ki-67 proliferation index was high (≥70%) [7]. A study by Guitarte et al. showed an unfavourable prognosis in these patients with an overall survival rate of 31% [8]. Therefore, GCC of the cervix is aggressive and prone to early metastasis. Early diagnosis, especially with smaller exophytic masses, improves prognosis. Treatment involves radical hysterectomy and adjuvant irradiation. Despite resembling other poorly differentiated carcinomas, its occurrence in younger patients emphasizes the need for early recognition and aggressive management.

Case 2: BSCC of the cervix (BSCC)

BSCC of the uterine cervix is an extremely rare malignant tumour of the female genital tract with a poorer clinical outcome than squamous cell carcinoma. Compared to our patient, Kwon et al. mentioned an elderly female, 70 years of age with vaginal bleeding whose pathologic diagnosis was BSCC [9]. In concordance with our patient, vaginal bleeding was the most typical symptom, as also observed by Salarvand et al. [10]. Histologically, these tumours exhibit an infiltrative growth pattern, small basaloid cells and prominent peripheral palisading [11]. The differential diagnosis of BSCC includes a solid variant of adenoid cystic carcinoma, which usually shows positivity for C-kit, SCNEC, which shows a positive reaction for TTF-1 and a negative reaction for 34βE12. Positive staining for 34βE12 excludes large cell neuroendocrine carcinoma. TTF-1 and 34βE12, associated with the specific neuroendocrine markers, are believed to be helpful panels of antibodies for differentiating basaloid carcinomas from other carcinomas with small cell morphology. Given their rarity, the diagnosis of pure BSCC is essentially essential due to its prognostic importance and biologically aggressive nature.

Cases 3 and 4: PSCC

PSCC of the uterine cervix may show transitional or squamous differentiation. It is a rare subtype that often resembles urothelial carcinoma of the urinary tract. A study of nine PSCCs of the cervix by Anand et al. had shown that patients usually are within an age range of 35-75 years and report to clinicians with per vaginal bleeding, as seen in our case [12].

Histologically, it exhibits complex papillary architecture, making superficial biopsies challenging for diagnosis, even if the invasion is not evident and atypia is moderate. Most cases display papillary structures with prominent fibrovascular cores, although micropapillary architecture without fibrovascular cores is rare and undocumented. Demonstrating invasion histologically can be difficult in superficial biopsies. A high index of suspicion is crucial for diagnosis. The intricate papillary architecture makes assessing tumour depth in excision specimens challenging. PSCC typically shows narrower, longer fibrovascular cores and more pronounced cytological atypia and pleomorphism than papillary immature metaplasia. PSCC is associated with high expression of Ki-67 and p53, suggesting significant proliferative activity and dysfunctional p53 due to HPV infection. HPV, particularly its E6 oncoprotein, binds to p53, leading to its degradation and dysfunction, similar to p53 mutation in other tumours.

The differentials include CIN3 with papillary configuration, condyloma, squamous papilloma, transitional cell carcinoma, papillary serous carcinoma and papillary adenocarcinoma. A study by Randall et al. has shown that late recurrences and metastases are common in PSCC, with positive CK7 and negative CK20 immunostaining and nuclear accumulation of mutant p53 observed [13]. These features distinguish it from other cervical carcinomas. Understanding these histological and molecular characteristics is essential for accurately diagnosing and managing PSCC, as they are highly aggressive malignant tumours.

Case 5: SCNEC of the cervix

SCNEC of the cervix is a rare cancer with high metastatic potential. Early diagnosis is essential for better patient outcomes. Symptoms usually include vaginal bleeding after sexual intercourse. The differentials include follicular cervicitis, lymphoma and metastatic carcinoma from the lung.

Since HPV infection is associated with cervical SCNEC, it is essential to screen for cervical precancerous lesions, primarily through TCT (ThinPrep cytology test) and HPV testing. HPV infection, specifically by subtype 18, is associated with the development of SCNEC. Prompt intervention at the pre-cancerous stage is crucial. An association with HPV was also seen in a study by Alejo et al., who found 85% of SCNEC cases to be HPV-related [14]. An analysis done on 19 cases of small cell carcinoma of the cervix by Lu et al. in 2022 highlights the significance of diagnosing this entity [15]. Cohen et al. also correlated the clinical importance of acknowledging this entity due to its high incidence of metastasis [16]. A study by Miyoshi et al. showed that the prognosis of SCNEC is poorer than that of other histological types of cervical carcinoma [17].

Case 6: USCC

USCC is a rare malignancy. The clinicopathological features of this entity need to be better understood due to the scarcity of these cases. Unlike its common occurrence in the ovary and fallopian tubes, USCC poses diagnostic challenges. Limited reports on HPV status in USCC are available; however, studies suggest its presence in a small fraction of cases. IHC is very helpful in the diagnosis and differentiation, and markers such as p53, p16, and CA125 are expressed in USCC and similar serous tumours. Although rare, understanding the characteristics and molecular features of USCC is important for diagnosis and treatment. In a study by Kitade et al., clinicopathological features were also compared with those of serous carcinomas of the endometrium and ovary [18].

A study by Kitade et al. showed the median age to be 54 years and the most common symptom to be vaginal bleeding, which is comparable to our patient under study [18]. Kitade et al. also noted that the cases showed positivity for ER, vimentin, and mutant p53, while PR was negative [18]. Togami et al. studied 12 cases of serous carcinoma of the cervix and saw that stage pT1b disease may have a relatively favourable outcome after radical surgery than those with advanced disease [19].

## Conclusions

This case series highlights the critical importance of identifying rare cervical carcinoma subtypes and variants. The detailed examination of these cases reveals unique clinicopathological features that can often be overlooked, leading to misdiagnosis and suboptimal treatment. IHC has proven vital in confirming these rare subtypes and their differentiation and identifying specific HPV associations. Further accumulation of the cases is warranted to clarify the disease's nature and behaviour, emphasising the necessity for continued research and education on these uncommon subtypes to advance our understanding and improve diagnostic accuracy. Recognizing and understanding the histopathological characteristics of these rare variants are essential steps towards better clinical outcomes and more effective management of cervical carcinoma.
